# A retrospective cohort study of 238,000 COVID-19 hospitalizations and deaths in Brazil

**DOI:** 10.1038/s41598-022-07538-0

**Published:** 2022-03-07

**Authors:** Marcos Felipe Falcão Sobral, Antonio Roazzi, Ana Iza Gomes da Penha Sobral, Brigitte Renata Bezerra de Oliveira, Gisleia Benini Duarte, Jadson Freire da Silva, Renata Maria Toscano Barreto Lyra Nogueira

**Affiliations:** 1 Programa de Pós-Graduação em Administração e Desenvolvimento, Universidade Federal Rural de Pernambuco, Avenida Dom Manoel de Medeiros, s/n – Dois Irmãos, Recife, PE Brazil; 2 Programa de Pós-Graduação em Psicologia Cognitiva, Universidade Federal de Pernambuco, Av. Prof. Moraes Rego, 1235 – Cidade Universitária, Recife, PE Brazil; 3 Universidade Federal de Pernambuco, Av. Prof. Moraes Rego, 1235 – Cidade Universitária, Recife, PE Brazil

**Keywords:** Health care, Epidemiology

## Abstract

The coronavirus disease (COVID-19) pandemic has overwhelmed health care systems in many countries and bed availability has become a concern. In this context, the present study aimed to analyze the hospitalization and intensive care unit (ICU) times in patients diagnosed with COVID-19. The study covered 55,563 ICU admissions and 238,075 hospitalizations in Brazilian Health System units from February 22, 2020, to June 7, 2021. All the patients had a positive COVID-19 diagnosis. The symptoms analyzed included: fever, dyspnea, low oxygen saturation (SpO2 < 95%), cough, respiratory distress, fatigue, sore throat, diarrhea, vomiting, loss of taste, loss of smell, and abdominal pain. We performed Cox regression in two models (ICU and hospitalization times). Hazard ratios (HRs) and survival curves were calculated by age group. The average stay was 14.4 days for hospitalized patients and 12.4 days for ICU patients. For hospitalized cases, the highest hazard mean values, with a positive correlation, were for symptoms of dyspnea (HR = 1.249; 95% confidence interval [CI], 1.225–1.273) and low oxygen saturation (HR = 1.157; 95% CI 1.137–1.178). In the ICU, the highest hazard mean values were for respiratory discomfort (HR = 1.194; 95% CI 1.161–1.227) and abdominal pain (HR = 1.100; 95% CI 1.047–1.156). Survival decreased by an average of 2.27% per day for hospitalization and 3.27% per day for ICU stay. Survival by age group curves indicated that younger patients were more resistant to prolonged hospital stay than older patients. Hospitalization was also lower in younger patients. The mortality rate was higher in males than females. Symptoms related to the respiratory tract were associated with longer hospital stay. This is the first study carried out with a sample of 238,000 COVID-19 positive participants, covering the main symptoms and evaluating the hospitalization and ICU times.

## Introduction

On December 31, 2019, the World Health Organization (WHO) was informed of several cases of pneumonia of unknown etiology, detected in several people in the city of Wuhan, China. In February 2020, the virus responsible for this condition was identified and named severe acute respiratory syndrome coronavirus 2 (SARS-CoV-2), which causes a disease known as coronavirus disease 2019 (COVID-19)^1^. On March 11, 2020, the WHO declared a public health emergency of international interest due to the high transmissibility of the virus^2^.

The outbreak likely started from consumption of contaminated animals or human contact with the Wuhan Seafood Market^3^. However, some researchers refute this theory because individuals from different continents who were not involved in the above situations were also infected with the virus^4^. A very recent investigation ordered by the US administration in May 2021, which could bring us closer to a definitive conclusion on the origins of the virus that has killed more than 4 million people globally and wrecked national economies, has reported inconclusive results on whether the virus jumped from animals to humans as part of a natural process or might have accidentally escaped from a Wuhan laboratory in central China^5,6^. Despite these doubts about the origin of the novel coronavirus, the most accepted explanation of transmissibility is that this virus spreads among humans through coughing, sneezing, and penetration of respiratory droplets or aerosols into the upper respiratory tract^4^. There are several ways in which coronaviruses are transmitted. The best-known routes are the direct route, that is, through direct contact with droplets and viral secretions, and the indirect route, from contact with environments contaminated with dirty water effluents, such as feces and urine^7,8^. As a few studies have reported that the direct route is primarily infectious and dominant for the dissemination of COVID-19^9,10^, other studies are currently investigating the indirect route as a source of aerosols associated with sewage or fomites. When viral particles reach the oral or nasal cavity, they spread and develop “intestinal tropism” or “pulmonary tropism,” the two preferential routes of this virus^10^.

The clinical manifestations of highly pathogenic human coronaviruses are not completely predictable as they range from asymptomatic infection to severe conditions. The lack of a determined standardization of the evolutionary or remissive symptoms of this disease has been one of the most intriguing issues associated with the pandemic. Cytokine storms and viral evasion of cellular immune responses are thought to play important roles in disease severity^11^.

Although little is known about the pathogenesis of any of the human coronaviruses (229E, OC43, HKU1, NL63, and SARS-CoV), there have been extensive studies on the pathogenesis of some animal coronaviruses, which have contributed to the understanding of human viruses^12,13^ (6F; 6G). Currently, it is expected that SARS-CoV-2 dysregulates the immune inflammatory response in a manner comparable to severe acute respiratory syndrome coronavirus 1 (SARS-CoV) and Middle East respiratory syndrome coronavirus (MERS-CoV). Severe COVID-19 is characterized by organ dysfunction, hypercytokinemia, and lymphopenia. Immune dysfunction in patients with COVID-19, including lymphopenia, reduced numbers of CD4^+^ T cells, and abnormal cytokine levels, is a common feature and may be a decisive factor associated with disease severity and worse outcomes^14,15^.

However, some factors are associated with a higher hospitalization rate, such as increased C-reactive protein level with high sensitivity, lymphocytopenia, and high myocardial rates^16^. Viral and genetic factors (comorbidities) should also be considered in the evolution of COVID-19 prognosis, without discarding the possibility that socioeconomic deprivation may be associated with worse outcomes^17^. In Barcelona, Spain, an ecological study found that the incidence of this disease is inversely proportional to the socioeconomic gradient^17^.

By August 2021, more than 200 million cases of COVID-19 had been registered worldwide since the novel coronavirus emerged in China in December 2019. However, the actual number is believed to be even higher because a large number of less severe or asymptomatic cases tend to remain undetected, despite intensified testing in many countries. Studies indicate that infected individuals can be asymptomatic or present with mild to severe reactions, leading to inadvertent infections^18,19^.

Although this clinical spectrum is broad, the Brazilian Ministry of Health and international studies indicate that the most common signs and symptoms are fever (temperature ≥ 37.8 °C), cough, respiratory distress, myalgia, and fatigue^20,21^. A study in China found that the triad of fever, cough, and dyspnea was present in 15% of those affected^22^. Symptoms such as hyposmia, anosmia, and ageusia should also be considered^23^. According to the WHO, 80% of people with COVID-19 have mild symptoms, 15% are hospitalized due to oxygen therapy, and 5% need interventions and monitoring in intensive care units (ICUs)^2^. The heterogeneity of this pandemic has led to inequality in the allocation of resources for the affected age groups^24^. The impact of the pandemic may be more significant in the elderly because of their health vulnerabilities^25^.

It is noteworthy that some symptoms are associated with a more favorable prognosis^26^, as is the case for most individuals with fever, cough, myalgia, or fatigue^27^. However, individuals who develop dyspnea and hypoxemia have a more reserved prognosis and are more likely to be hospitalized and admitted to the ICU^26^. Regarding the time from symptom onset to clinical outcome, there is evidence that the time between symptom onset and hospital admission varies from 6 to 14 days, symptom onset to ICU admission varies from 8 to 15 days, symptom onset to death varies from 11 to 18 days, and symptom onset to hospital discharge can take up to 22 days^28,29^.

Hospital stay may vary under different health services due to individual peculiarities and recognizing the symptoms, or a set of symptoms, that exist in the affected population, allowing us to fill in existing gaps about COVID-19. Identifying the estimated length of stay, groups of symptoms expected from each age group, number of days elapsed from the onset of symptoms to hospitalization, and outcomes allows for directing management strategies for the care of the population. Furthermore, the hospitalization period can be modified in the context of intervention management, because the effectiveness of hospital care can influence mortality.

The impact of COVID-19 has created specific challenges for health systems of countries. Worldwide, the increase in hospital demands has been clearly seen with high hospitalizations, increased ICU bed requirements, increased use of more complex equipment and techniques such as mechanical ventilation, and a greater demand for intensive monitoring and care from qualified professionals^30^.

The Brazilian Unified Health System (SUS) must guarantee, through constitutional legislation, full and unrestricted medical access for the entire population of the country, with universality and equity being two of the guiding principles of individual care. Although a resurgence in the number of admissions has been observed, data on risk factors and mortality rates in hospitalized patients are scarce^31^. Regarding mortality data in the Brazilian SUS, a health care system with strained and tense medical staff, regional differences in access to resources, and overburdened hospital systems has created an unfavorable condition that has been further aggravated by recent economic and political crises. This has strengthened structural problems in the public health system and contributed to significantly more in-hospital deaths. Furthermore, even the perception of a strained health system can lead to excess mortality from COVID-19 and other conditions because individuals may avoid seeking care until their clinical condition has deteriorated or they may not even seek medical care and die at home.

The COVID-19 pathogenesis is still not completely defined and the intense pressure imposed on hospitals has led to care limitations for the most fragile patients. The present study uses data from a database available from the Brazilian Ministry of Health, updated by national coverage data, which allows us to perform comprehensive epidemiological analyses. Our study is the first to incorporate data on all COVID-19 hospitalizations in the Brazilian SUS. We covered the parameters of age group (eight groups covering age subgroups from 0 to > 85 years), main symptoms, and hospitalization or ICU time. Therefore, this study aimed to analyze the relationship between hospitalization or ICU time and COVID-19 deaths in Brazil. This was a retrospective study based on the secondary database available from the Brazilian Ministry of Health between February 22, 2020, and June 7, 2021. The database covers national centers and multicenter records that link with the Brazilian SUS.

## Methods

### Ethical approval

Our study used information from a public database made available by the Brazilian government through the DATASUS platform. All data in this database were processed by the Brazilian government, removing all identifiers.

The research was not sent to the Research Ethics Committee as it was covered by Article 1, Sole Paragraph, subparagraph II and III of RESOLUTION No. 510 of APRIL 7, 2016, of the Brazilian National Health Council: "Sole paragraph: They will not be registered or evaluated by the research ethics committee system: II—research using publicly accessible information, according to Law No. 12,527 of November 18, 2011; III—research using public domain information. The resolution is available at http://conselho.saude.gov.br/resolucoes/2016/Reso510.pdf. All methods were performed in accordance with the relevant guidelines and regulations.

### Study design, setting, data sources, and participants

We performed a cohort, retrospective, national, multicenter, analytical study with secondary data from the Brazilian Database of Severe Acute Respiratory Syndrome (SARS), which includes cases of COVID-19^32^ acquired through the Ministry of Health website. The information contained in this database is updated daily. The study was conducted in accordance with the Strengthening the Reporting of Observational Studies in Epidemiology guidelines.

In this study, the COVID-19 database notifications from February 22, 2020, until June 7, 2021, were used. The database comprises 639,405 patients placed in 6,124 sentinel units (SUs) of Brazil, distributed throughout 2,715 Brazilian cities. The SUs are physical units and working groups created to evaluate and conduct epidemiological assessments^33^. Patients admitted to care units for the treatment of COVID-19, with a final diagnosis of COVID-19 confirmed through laboratory, epidemiological, and imaging tests, were included.

### Variables

The following 12 symptoms were the independent variables: fever, dyspnea, low oxygen saturation (SpO_2_ < 95%), cough, respiratory distress, fatigue, sore throat, diarrhea, vomiting, loss of taste, loss of smell, and abdominal pain. A categorical variable of eight age groups was also added, following the pattern adopted by the Centers for Disease Control and Prevention^34^: 0–17, 18–29, 30–39, 40–49, 50–64, 65–74, 75–84, and ≥ 85 years. This variable was calculated based on a patient's age at admission.

### Study size and missing data

We used predictors for two outcomes: hospitalization and ICU time (days). The database contained 639,405 records. Hospitalization time was calculated as the difference between the hospitalization and discharge dates. For this model, the sample size was 238,075 records, 401,330 records were excluded because of missing data points. This missing data included a lack of filled-in data, such as the patient's admission or discharge dates.

ICU time is the interval between ICU entrance and discharge. The sample size was 55,562 records, with 583,842 records excluded because of missing data and a lack of ICU admission.

### Statistical analysis methods

We analyzed hospitalization and ICU time in days. Thus, we performed two models, the first with hospitalization time and the second with ICU time.

**Figure 1:**
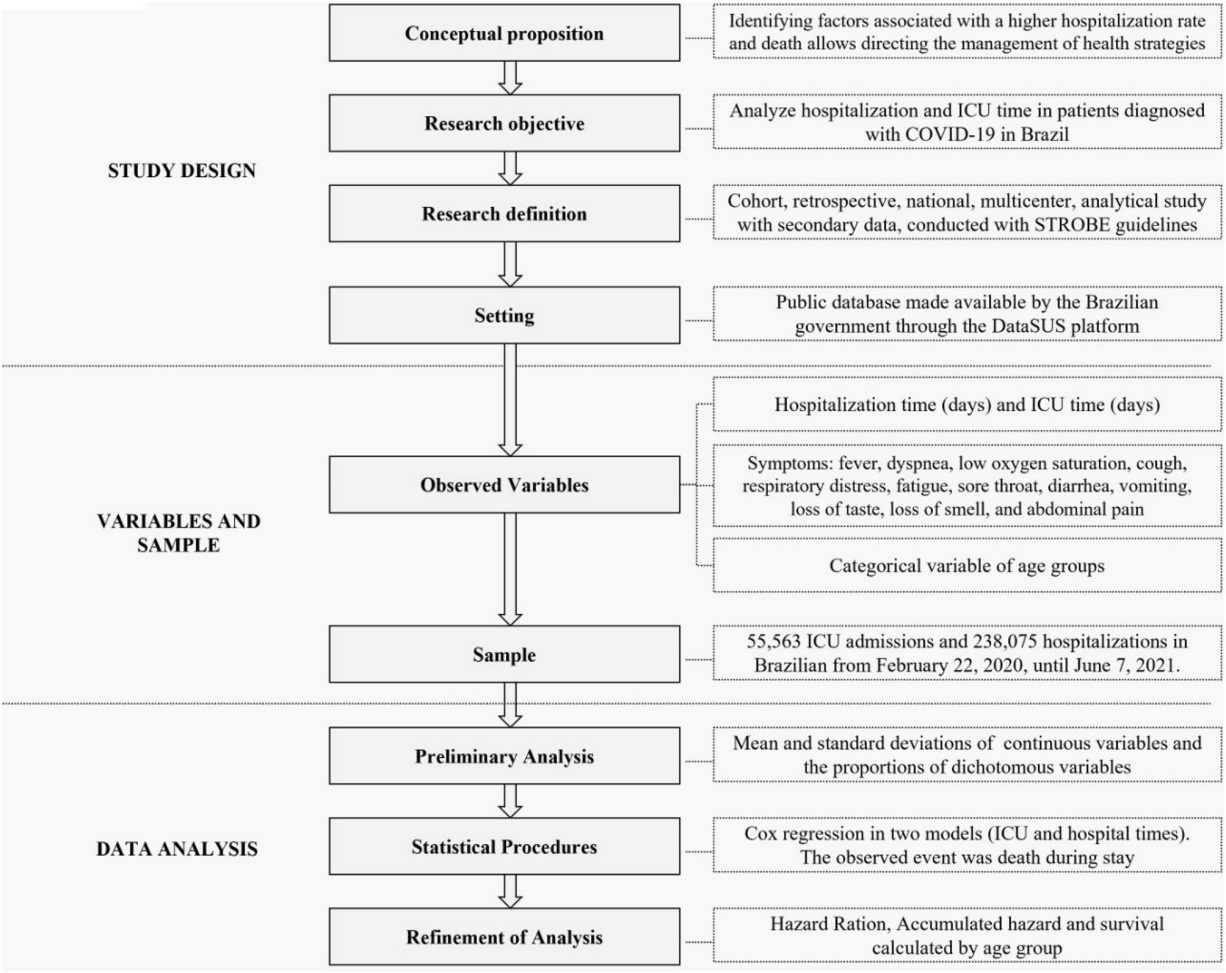
Study design.

We report continuous variables with means and standard deviations. We calculated the proportions of dichotomous variables. Thereafter, Cox regression was calculated (hospitalization and ICU time), using the enter method and IBM SPSS Statistics 23 (https://www.ibm.com/support/pages/downloading-ibm-spss-statistics-23). The observed event was death during hospitalization or ICU stay. All variables with *P* > 0.05 were excluded from the analysis. We calculated hazard and survival functions for each age group. The mean coefficients of the equation, survival, and hazard for each independent variable were analyzed. We also calculated the cumulatedhazard. It indicates how the death risk increases or decreases depending on hospitalization or ICU time. In this way, we measure how high or low the death risk is while the patient remains hospitalized. The cumulated hazard demonstrates that more days hospitalized can represent an increased or reduced risk of death. The study design is shown in Fig. 1.

## Results

Table 1 shows the data summary of patients, recoveries, and deaths. This retrospective analytical cohort study identified factors and mortality risks that interfere with the length of hospital and ICU stay in individuals with COVID-19. Of the 238,075 hospitalized patients, 161,150 (68%) recovered, while 76,925 (32%) died.

**Table 1:** Data summary.

	Patients	Recoveries	Deaths
ICU	55,563	23,907	31,656
Hospitalization	238,075	161,150	76,925

The mean hospitalization time among those who recovered was 10.79 ± 15.28 days, while that of those who died was 14.47 ± 15.18 days. Men constituted the majority of hospitalized patients with 132,639 (56%) cases, while 105,396 (44%) patients were women. The proportion of deaths was higher in men (33%) than women (30%). The average hospitalization days was 11.66 ± 15.19 days for women and 9.70 ± 7.06 days for men. The hospitalization time in elderly patients was longer than that in young people. The average number of days for hospitalization/ ICU admission, by age group, were: 0–17 (9.15 ± 16.00 days), 18–29 (9.23 ± 14.74 days), 30–39 (9.42 ± 14.02 days), 40–49 (10.48 ± 15.42 days), 50–64 (12.06 ± 14.77 days), 65–74 (13.69 ± 16.31 days), 75–84 (13.20 ± 15.94 days), and ≥ 85 (11.61 ± 13.93 days).

A total of 55,563 cases of ICU admission were analyzed; 23,907 (43%) patients recovered and 31,656 (57%) patients died. Patients who recovered remained in the ICU for 10.69 ± 12.36 days, while those who died remained for 12.84 ± 13.37 days. As for demographic parameters, most of those admitted to the ICUs were men, with 32,277 (58.7%) male cases and 23,286 (41.9%) female cases. Deaths were also higher in men than women. Women (11.62 ± 12.89) stayed in ICUs for less time than men (12.13 ± 13.06). The average ICU times in days by age group were: 0–17 (9.52 ± 14.78 days), 18–29 (9.38 ± 12.33 days), 30–39 (10.11 ± 11 0.42 days), 40–49 (11.19 ± 12.61 days), 50–64 (12.69 ± 13.31 days), 65–74 (13.33 ± 13.98 days), 75–84 (11.50 ± 12.48 days), and ≥ 85 (9.37 ± 10.59 days).

We found that 92.3% of patients had up to seven symptoms during hospitalization and the mean number of symptoms was 4.58 ± 1.96. The symptoms, ordered in decreasing order of frequency, were cough (70.4%), dyspnea (73%), SpO2 < 95% (64%), respiratory discomfort (61.6%), fever (59.2%), fatigue (25.1%), sore throat (18.1%), diarrhea (15.3%), loss of taste (12.1%), loss of smell (12%), vomiting (8.8%), and abdominal pain (6.6%). In the ICU, 92% of patients had up to seven symptoms and the mean number of symptoms was 4.73 ± 1.95. The most frequent symptoms were dyspnea (81%), SpO2 < 95% (74.4%), respiratory discomfort (68.4%), cough (68.1%), fever (58.4%), fatigue (25%), sore throat (15.8%), diarrhea (13.7%), loss of smell (10%), loss of taste (9.9%), vomiting (7.8%), and abdominal pain (5.9%).

### Hospitalization time

The hospitalization time covered 238,075 records, which included 161,150 censored, and 76,925 valid events. The cases by age group included the following: 0–17 (4,181 cases), 18–29 (7,793 cases), 30–39 (19,947 cases), 40–49 (30,999 cases), 50–64 (68,358 cases), 65–74 (50,731 cases), 75–84 (37,206 cases), and ≥ 85 (18,860 cases). Sore throat, vomiting, and loss of smell were not statistically significant. The variables that positively correlated with hospitalization time were dyspnea (hazard ratio [HR] = 1.249; 95% confidence interval [CI], 1.225–1.273), SpO2 < 95% (HR = 1.157; 95% CI 1.137–1.178), vomiting (HR = 1.012; 95% CI 0.984–1.041), and abdominal pain (HR = 1.055; 95% CI 1.023–1.089). Fever, cough, diarrhea, fatigue, and loss of taste were negatively correlated. The Cox regression analysis is shown in Table 2.

**Table 2:** Cox regression analysis (hospitalization time).

Symptom	B	Wald	Sig	HR
Fever	−0.041	28.847	0	0.960
Cough	−0.128	257.770	0	0.880
Sore throat	−0.002	0.024	0.877	0.998
Dyspnea	0.222	510.727	0	1.249
Respiratory distress	0.241	760.607	0	1.273
SpO2 < 95%	0.146	259.635	0	1.157
Diarrhea	−0.100	76.032	0	0.905
Vomiting	0.012	0.717	0.397	1.012
Fatigue	−0.068	58.234	0	0.934
Abdominal pain	0.054	11.136	0.001	1.055
Loss of smell	−0.024	1.541	0.215	0.977
Loss of taste	−0.098	26.567	0.000	0.907
Age		11,613.647	0	
Age (1)	−1.902	945.448	0	0.149
Age (2)	−1.513	1590.265	0	0.220
Age (3)	−1.453	3738.530	0	0.234
Age (4)	−1.233	5036.218	0	0.291
Age (5)	−0.914	5748.528	0	0.401
Age (6)	−0.587	2558.663	0	0.556
Age (7)	−0.315	720.393	0	0.730

**Figure 2:**
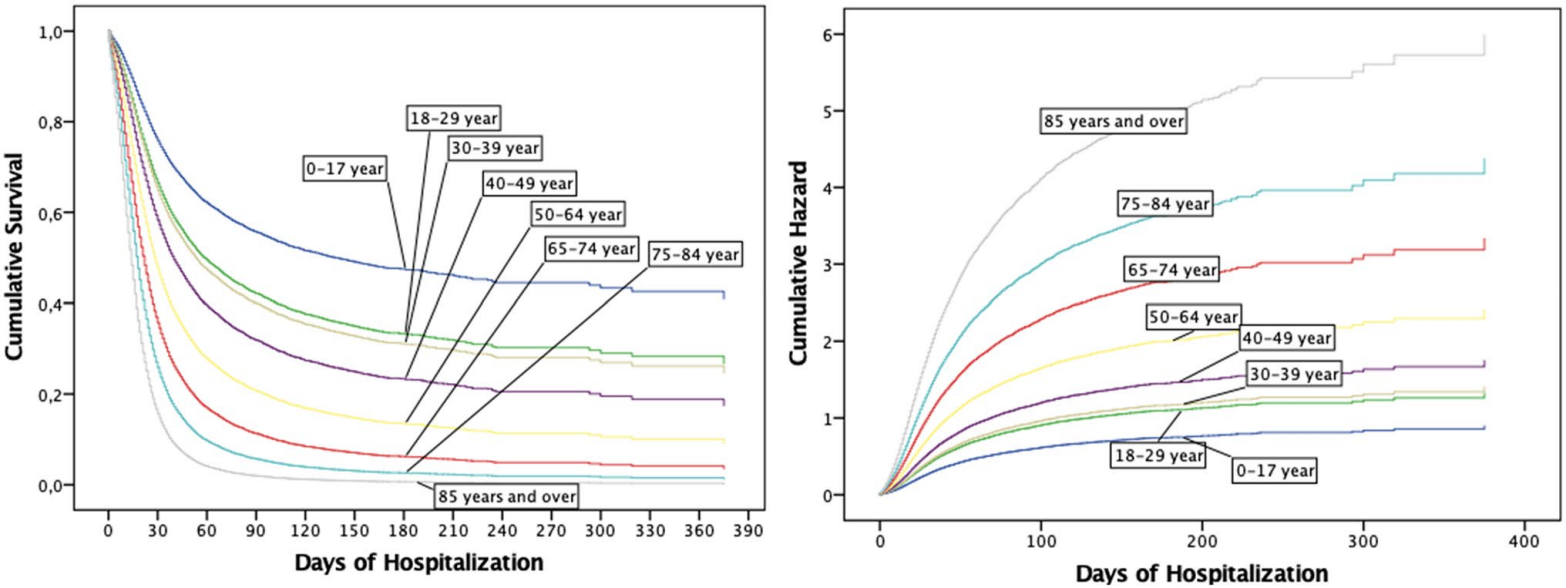
Cumulative survival (left panel) and cumulative hazard (right panel)” during Hospitalization time.

The hospitalization time showed a symmetrical pattern by age group, demonstrating that younger people, compared to the elderly, have a lower average risk of mortality due to the length of hospital stay. The hazard and survival cumulative functions are shown in Fig. 2.

The average survival by age group was as follows: 0–17 (0.94 ± 0.09 days), 18–29 (0.92 ± 0.11 days), 30–39 (0.91 ± 0.11 days), 40–49 (0.87 ± 0.14 days), 50–64 (0.80 ± 0.19 days), 65–74 (0.72 ± 0.23 days), 75–84 (0.67 ± 0.25 days), and ≥ 85 (0.63 ± 0.26 days).

Over 22 days, the survival probability of hospitalized patients dropped by an average of 2.27% per day. After this period, patients had less than 50% survival and the cumulatedhazard was 8.11. For older adults, the probability of survival reached 50% after the thirteenth day. On the twenty-third day, patients aged ≥ 85 years had a survival rate of 24%. The survival and cumulative hazard by age group and day are shown in Table 3.

**Table 3:** Hospitalization survival and hazard by age group.

	Survival by age group									Cumulative hazard by age group									
Day	1	2	3	4	5	6	7	8	All	1		2	3	4	5	6	7	8	All
0	1.00	0.99	0.99	0.99	0.99	0.99	0.98	0.97	0.99	0.00		0.01	0.01	0.01	0.01	0.02	0.02	0.03	0.01
1	0.99	0.99	0.99	0.98	0.98	0.97	0.96	0.94	0.97	0.01		0.02	0.02	0.02	0.03	0.05	0.07	0.09	0.04
2	0.99	0.98	0.98	0.98	0.97	0.95	0.93	0.91	0.96	0.02		0.03	0.04	0.05	0.07	0.10	0.13	0.19	0.09
3	0.98	0.98	0.97	0.97	0.95	0.93	0.91	0.88	0.95	0.04		0.06	0.07	0.08	0.12	0.17	0.23	0.32	0.14
4	0.98	0.97	0.96	0.96	0.94	0.91	0.89	0.84	0.93	0.06		0.09	0.10	0.13	0.18	0.26	0.35	0.49	0.22
5	0.97	0.96	0.96	0.94	0.92	0.89	0.86	0.81	0.91	0.09		0.14	0.15	0.19	0.26	0.37	0.50	0.70	0.31
6	0.97	0.95	0.95	0.93	0.91	0.87	0.83	0.77	0.89	0.12		0.19	0.20	0.26	0.36	0.51	0.69	0.96	0.43
7	0.96	0.94	0.94	0.92	0.89	0.85	0.80	0.74	0.87	0.17		0.25	0.27	0.34	0.48	0.68	0.91	1.27	0.57
8	0.95	0.93	0.92	0.90	0.87	0.82	0.77	0.69	0.84	0.21		0.32	0.35	0.44	0.62	0.88	1.17	1.64	0.74
9	0.94	0.92	0.91	0.89	0.85	0.80	0.74	0.66	0.82	0.27		0.41	0.44	0.56	0.78	1.11	1.48	2.06	0.95
10	0.94	0.91	0.90	0.87	0.83	0.77	0.70	0.62	0.79	0.34		0.51	0.55	0.69	0.97	1.37	1.84	2.55	1.20
11	0.93	0.89	0.89	0.86	0.81	0.74	0.67	0.57	0.76	0.41		0.62	0.67	0.85	1.19	1.68	2.24	3.11	1.48
12	0.92	0.88	0.87	0.84	0.79	0.71	0.64	0.54	0.73	0.50		0.74	0.81	1.03	1.43	2.02	2.69	3.74	1.80
13	0.91	0.87	0.86	0.83	0.76	0.68	0.60	0.51	0.70	0.59		0.88	0.96	1.22	1.71	2.41	3.21	4.43	2.17
14	0.90	0.85	0.84	0.81	0.74	0.66	0.57	0.47	0.68	0.70		1.05	1.13	1.43	2.01	2.83	3.77	5.20	2.57
15	0.89	0.85	0.83	0.79	0.72	0.63	0.55	0.43	0.65	0.82		1.21	1.32	1.67	2.34	3.30	4.38	6.05	3.02
16	0.88	0.83	0.82	0.77	0.70	0.60	0.51	0.41	0.63	0.95		1.40	1.52	1.93	2.70	3.80	5.05	6.96	3.51
17	0.87	0.82	0.80	0.75	0.68	0.58	0.49	0.38	0.60	1.08		1.60	1.75	2.22	3.10	4.36	5.78	7.94	4.05
18	0.87	0.80	0.78	0.74	0.66	0.55	0.46	0.35	0.58	1.22		1.83	1.99	2.52	3.52	4.96	6.57	9.01	4.62
19	0.85	0.79	0.77	0.72	0.63	0.53	0.43	0.32	0.56	1.39		2.06	2.25	2.84	3.98	5.61	7.42	10.17	5.24
20	0.82	0.78	0.76	0.71	0.62	0.51	0.41	0.31	0.54	1.59		2.31	2.53	3.19	4.47	6.29	8.34	11.38	5.90
21	0.83	0.76	0.75	0.69	0.60	0.49	0.39	0.29	0.53	1.78		2.59	2.83	3.56	4.99	7.02	9.30	12.67	6.59
22	0.81	0.75	0.73	0.68	0.58	0.47	0.37	0.26	0.50	1.99		2.88	3.14	3.95	5.54	7.79	10.31	14.07	7.34
23	0.81	0.74	0.72	0.67	0.56	0.45	0.35	0.24	0.49	2.19		3.17	3.47	4.35	6.12	8.60	11.38	15.54	8.11

Note: 1: 0–17, 2: 18–29, 3: 30–39, 4: 40–49, 5: 50–64, 6: 65–74, 7: 75–84, and 8: ≥ 85 years.

### ICU time

The ICU survival model included 55,563 admissions records of which, 23,907 were censored and 31,656 were valid. The number of cases by age group were as follows: 0–17 (594 cases), 18–29 (1,163 cases), 30–39 (3,352 cases), 40–49 (5,810 cases), 50–64 (15,464 cases), 65–74 (13,778 cases), 75–84 (10,540 cases), and ≥ 85 (4,862 cases). Of the 12 variables related to symptoms, SpO2 < 95%, loss of smell, and loss of taste were excluded for presenting p-values > 5%.

Respiratory distress showed a positive correlation in the ICU and the presence of this symptom had an HR of 1.194 (95% CI 1.161–1.227). Abdominal pain (HR = 1.100; 95% CI 1.047–1.156), dyspnea (HR = 1.094; 95% CI 1.059–1.130), and vomiting (HR = 1.050; 95% CI 1.005–1.097) were also positively correlated. Due to negative correlations, fever, cough, respiratory distress, diarrhea, and fatigue are related to protective factors. The Cox regression analysis is shown in Table 4.

**Table 4:** Cox Regression Analysis (ICU).

Symptom	B	Wald	Sig	HR
Fever	−0.067	32.119	0	0.935
Cough	−0.067	28.694	0	0.935
Sore throat	0.034	4.426	0.035	1.035
Dyspnea	0.09	29.873	0	1.094
Respiratory distress	0.177	157.838	0	1.194
SpO2 < 95%	−0.015	0.965	0.326	0.985
Diarrhea	−0.049	7.625	0.006	0.953
Vomiting	0.049	4.721	0.03	1.05
Fatigue	−0.053	15.235	0	0.948
Abdominal pain	0.095	14.262	0	1.1
Loss of smell	−0.017	0.361	0.548	0.983
Loss of taste	−0.005	0.025	0.875	0.995
Age		2832.895	0	
Age (1)	−1.538	229.819	0	0.215
Age (2)	−1.019	310.773	0	0.361
Age (3)	−1.072	874.297	0	0.342
Age (4)	−0.97	1242.017	0	0.379
Age (5)	−0.772	1489.314	0	0.462
Age (6)	−0.542	776.956	0	0.582
Age (7)	−0.27	185.597	0	0.764

**Figure 3:**
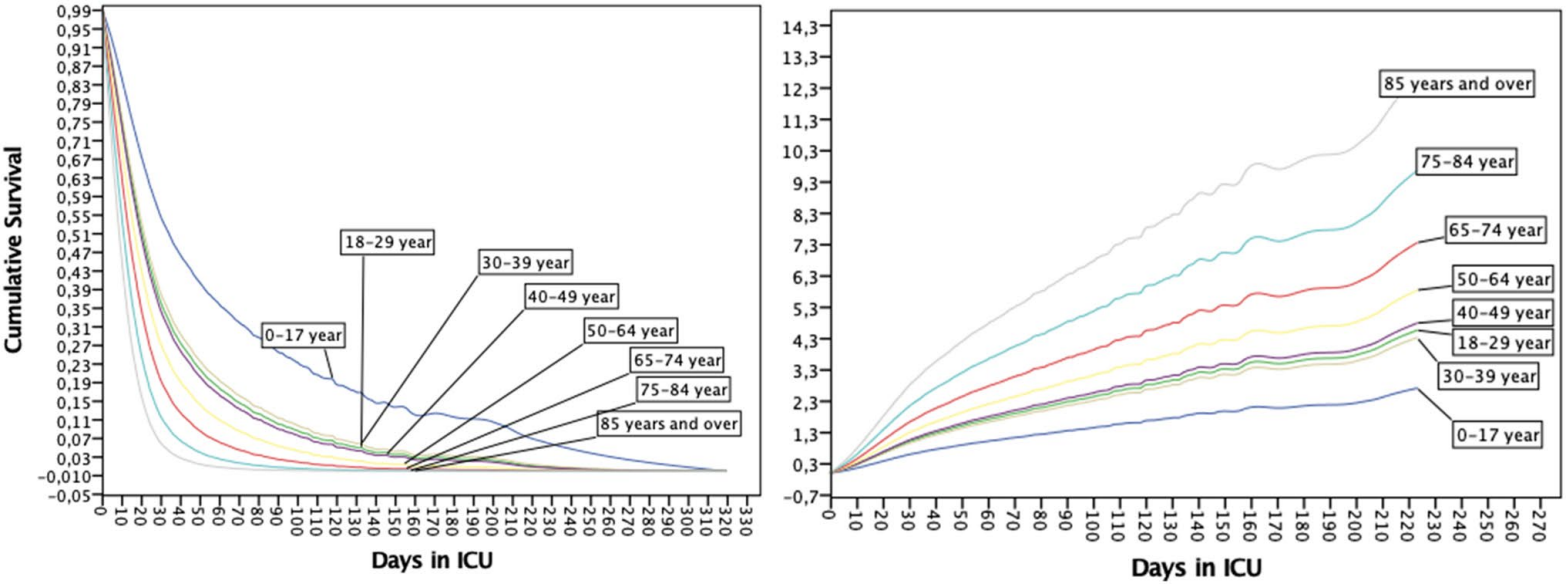
Cumulative survival (left panel) and cumulative hazard (right panel)” during ICU Time.

Survival curves showed distinct patterns between age groups. In the ICU, the probability of death depends on the patient's age. Younger patients were more resistant to prolonged hospital stays than elderly patients. Despite this, the 30–39 years age group had better hazard and survival curves than the 18–29 years age group. The cumulative hazard and survival curves are shown in Fig. 3.

The average survival by age group was as follows: 0–17 (0.87 ± 0.14 days), 18–29 (0.85 ± 0.16 days), 30–39 (0.80 ± 0.18 days), 40–49 (0.74 ± 0.21 days), 50–64 (0.66 ± 0.24 days), 65–74 (0.59 ± 0.26 days), 75–84 (0.58 ± 0.27 days), and ≥ 85 (0.58 ± 0.27 days).

The daily mean survival probability decreased by 3.27% per day during the first 15 days. Patients who stayed for more than 14 days in the ICU had less than 50% probability of survival. Survival declined more sharply depending on age group. On the tenth day, patients aged ≥ 85 years had a 50% chance of survival. The survival and cumulative hazard by age group and day are shown in Table 5.

**Table 5:** ICU survival and hazard by age group.

	Survival by age group									Cumulative hazard by age group								
Day	1	2	3	4	5	6	7	8	All	1	2	3	4	5	6	7	8	All
0	0.99	0.99	0.99	0.98	0.98	0.97	0.97	0.96	0.97	0.01	0.01	0.01	0.02	0.02	0.03	0.03	0.04	0.03
1	0.98	0.97	0.97	0.96	0.95	0.94	0.92	0.90	0.94	0.03	0.04	0.05	0.06	0.07	0.09	0.12	0.15	0.09
2	0.97	0.96	0.95	0.94	0.92	0.90	0.88	0.85	0.91	0.06	0.08	0.10	0.12	0.15	0.19	0.25	0.31	0.19
3	0.96	0.95	0.93	0.92	0.89	0.87	0.84	0.80	0.88	0.11	0.13	0.17	0.21	0.27	0.34	0.43	0.53	0.32
4	0.94	0.93	0.91	0.89	0.86	0.83	0.80	0.75	0.84	0.17	0.21	0.26	0.33	0.41	0.52	0.65	0.83	0.49
5	0.93	0.91	0.89	0.87	0.83	0.80	0.75	0.70	0.81	0.24	0.30	0.37	0.47	0.59	0.75	0.94	1.19	0.71
6	0.92	0.90	0.87	0.84	0.80	0.76	0.71	0.65	0.78	0.33	0.41	0.51	0.64	0.81	1.02	1.29	1.63	0.97
7	0.90	0.88	0.85	0.82	0.77	0.72	0.66	0.60	0.74	0.43	0.54	0.67	0.84	1.07	1.35	1.70	2.14	1.28
8	0.89	0.86	0.83	0.79	0.74	0.68	0.62	0.55	0.70	0.55	0.69	0.86	1.08	1.37	1.73	2.18	2.74	1.64
9	0.86	0.84	0.80	0.76	0.71	0.65	0.58	0.51	0.66	0.70	0.86	1.08	1.35	1.71	2.17	2.73	3.42	2.06
10	0.86	0.82	0.78	0.73	0.68	0.61	0.54	0.47	0.63	0.85	1.07	1.32	1.66	2.10	2.66	3.34	4.18	2.54
11	0.84	0.80	0.76	0.71	0.65	0.58	0.50	0.42	0.60	1.03	1.29	1.59	2.01	2.53	3.21	4.02	5.06	3.06
12	0.82	0.78	0.74	0.68	0.62	0.55	0.47	0.39	0.57	1.23	1.53	1.90	2.39	3.01	3.81	4.79	6.02	3.65
13	0.80	0.76	0.71	0.66	0.59	0.52	0.43	0.35	0.54	1.45	1.80	2.23	2.80	3.54	4.48	5.62	7.07	4.29
14	0.79	0.75	0.69	0.63	0.56	0.48	0.40	0.32	0.50	1.69	2.09	2.60	3.27	4.12	5.22	6.54	8.23	5.00
15	0.77	0.72	0.67	0.60	0.53	0.45	0.37	0.29	0.48	1.95	2.42	3.00	3.78	4.76	6.01	7.55	9.48	5.77

Note: 1: 0–17, 2: 18–29, 3: 30–39, 4: 40–49, 5: 50–64, 6: 65–74, 7: 75–84, and 8: ≥ 85 years.

## Discussion

COVID-19 infects individuals from all age groups. The transmission and its consequences may vary according to individual biological factors, environmental and socioeconomic characteristics, public policies developed, and the health system's capacity^35^. Several studies have described factors contributing to transmission, clinical presentation, mortality, possible treatments, and short-term results^26^. Some signs and symptoms can be interpreted as risk factors, indicating the need for hospitalization or transfer to the ICU.

Many COVID-19 symptoms are similar to those of seasonal influenza. However, Piroth et al.^36^ reported some prominent differences, namely, a higher rate and length of hospitalization, gender and age groups most affected, need for invasive mechanical ventilation, length of stay in the ICU, and mortality rate.

In the first reports of COVID-19, age group was an identified risk factor. Older people were more affected, had more extended hospital stays, greater clinical severity, and high mortality^18,37^. Our results also indicated that older age groups were associated with extended hospital and ICU times. This may be related to the vulnerability of this population^38^, which includes immunosenescence and changes in the respiratory system, such as decreased respiratory capacity and production of surfactants^39^. Our results also converge with some studies^35,37,40^ by showing that the population most affected by COVID-19 is between 50 and 64 years of age. Recently, a worldwide survey identified that approximately 72% of confirmed cases of SARS-CoV-2 infection were aged ≥ 40 years^41^. In addition to age, comorbidities are another factor associated with hospitalization and ICU time, such as cardiovascular diseases, hypertension, diabetes, chronic respiratory diseases^42^, and smoking status^43^. We did not include comorbidities in our study, but it is noteworthy that after age 50, 67.8% of people have multimorbidity^44^. During the initial epidemic in Hubei, Gao^45^ reported that 58% of patients admitted to the ICU had at least one comorbidity, which increased the probability of progression to severe forms of the disease.

In addition to high age group, many studies identified that affected individuals were predominantly male^45^ and that the mortality rate was also higher in this population^46^. Research has identified that males were affected most in the SARS and MERS epidemics^47,48^. Thus, the results presented in this study converge with these findings, considering the predominance of males in hospitalization and ICU admission and mortality. It has been reported that differences in the levels and types of male and female sex hormones influence the susceptibility to infection by COVID-19 because sex hormones modulate adaptive and innate immune responses^49^. Thus, the reduced susceptibility of females to viral infections can be attributed to the protection from the X chromosome and sex hormones^50^, especially estrogen^46^.

In terms of identifying percentage changes in mortality rate due to specific comorbidities, Wu and McGoogan^51^ identified a 10.5% increased mortality rate in individuals with cardiovascular diseases, 7.3% for those with diabetes, 6.3% for those with chronic respiratory diseases, and 6% for those with hypertension. In concordance with the information prior, we identified that respiratory symptoms, such as respiratory discomfort, dyspnea, and SpO2 < 95%, correlated positively with length of stay, abdominal pain, and vomiting.

In Lombardy (Italy), the epicenter of the first outbreak of COVID-19 in a Western country, respiratory syndrome was present in 40% to 96% of ICU patients^31^. However, a study^52^ highlighted that the most common and persistent symptoms in cases of long-term COVID-19 in patients aged 5 to 17 years were fatigue, headache, and loss of smell. Our study identified cough, fever, and respiratory discomfort/decompensation as the most common symptoms in cases of both hospitalization and ICU admission.

Concerning ICU time and survival, Blake et al.^53^ found that the mean time interval from the first report of symptom onset to admission to the ICU was 10 days and, with respiratory decompensation, invasive mechanical ventilation was required in almost 75% of cases, resulting in intubation in 76% of these individuals after the first 24 h after admission to the ICU. Research in China identified that 39% of patients hospitalized for 28 days died and 97% of individuals who needed mechanical ventilation had the same outcome^31^.

Our study identified that survivors spent 10 days hospitalized, while death occurred 14 days after admission. In addition, we found that patient survival decreased by 2.27% per day of hospitalization. The larger chance of death during hospitalizations occurred after 10–14 days in the ICU. The most significant resistance to death was observed in younger patients. Our results agree with the findings in the literature that indicates that survival for older adults reaches 50% after the thirteenth day of hospitalization.

Some researchers have suggested that COVID-19 inhibits the body's cellular immune function, causing damage to T lymphocytes^54,55^. Thus, the low absolute value of lymphocytes could be used as an evaluative index or a reference score in the diagnosis of new COVID-19 infections in clinic settings.

However, the hospitalization of patients, especially older adults, or people with comorbidities, who contract COVID-19 is one of the signs of disease severity as approximately 7.2% of hospitalized patients die. Similarly, the need to be admitted to the ICU has become a highly relevant means of assessing the impact of COVID-19 on public health worldwide.

The average length of hospitalization and ICU stay may have been influenced by the readiness of the public health service in Brazil. At the beginning of the pandemic period, especially during the first wave, there was reduced availability of beds and tests for the Brazilian population, which may have contributed to the delay in the treatment of patients and compromised the health system’s effectiveness.

## Conclusions

The heterogeneity of both the combination and severity of COVID-19 symptoms varies individually, hindering diagnosis and efficient clinical management. Identifying symptomatological patterns and clinical outcomes can guide more consistent approaches for reducing death and sequelae. The study presented a pattern between patient age, symptom groups, and hospital stay.

The results showed that symptoms associated with the respiratory system, namely dyspnea and SpO2 < 95%, were positively associated with a more extended total hospital stay. There was a positive correlation between respiratory discomfort and abdominal pain with respect to a patient's time in the ICU. We emphasize that a patient's probability of survival decreases significantly with each day of hospitalization, with a high chance of death when patients spend more than 14 days in the ICU or 20 days in the hospital, with younger patients showing better resistance. These data bring together potential warning symptoms for developing severe or critical conditions due to COVID-19 infection during the pandemic. Signs or symptoms suggestive of respiratory distress are associated with more severe disease progression and a worse prognosis. These conditions become latent if associated with age. Another relevant clinical scenario is the association between respiratory and digestive symptoms, especially abdominal pain. Intestinal involvement has been identified in autopsies and biopsies of patients with COVID-19 and some clinical reports have described abdominal discomfort as a primary symptom. These data can serve as a warning for the Delta and Ômicron variants of COVID-19. That is, patients with digestive symptoms, even without respiratory symptoms, should receive greater attention, especially if they report a history of epidemiological exposure.

Information was collected from a database provided by the Ministry of Health, Brazil. Since February 2020, the health crisis caused by the pandemic has highlighted the problems arising from investment in public health, which has undergone successive cuts in recent years. The different clinical manifestations and outcomes emphasize the importance of adhering to hygienic and preventive principles and highlight the importance of finding and developing new diagnostic approaches and therapeutic options sensitive to the action of SARS-CoV-2. In the current context of a global pandemic and public health emergency caused by COVID-19, knowledge of the protective and risk factors for developing COVID-19 infection or developing severe forms of the disease are essential for the proper management of patients in the early stages of the disease.

This study had limitations; the set of variables used did not encompass all variables associated with hospitalization and ICU time. Furthermore, the high proportion of missing data (63%) was a limitation of the routine national surveillance system. In addition, the research reflects the behavior of a specific country in COVID-19 treatment, with no statistical analysis performed to show a causal relationship between the variables of interest.

Future research must be conducted to broaden the discussion on the relationship between COVID-19 parameters and variables, such as prior comorbidities and sociodemographic characteristics of the population, to evaluate their impact on the patients’ time in hospital. Moreover, researchers must discuss the formation of public policies that prioritize democratic and equal access to health care systems, especially in countries like Brazil that have been through substantial economic and political instability, which have been worsened by the present health crisis. Health equity remains a significant challenge for policymakers and academics in this area. A recent challenge is to reduce mortality arising from new COVID-19 variants that continue to emerge across the globe, making it necessary for surveillance to identify them and studies to determine their transmissibility and severity. Finally, it is necessary to adopt public policies aimed at flattening the COVID-19 curve, which requires a combination of strategies to slow the spread of COVID-19 and spread out the epidemic's peak, preventing hospitals from reaching capacity and mortality.

## Availability of supporting data

The datasets used and analyzed during the current study are available from the corresponding author on reasonable request. The datasets generated and/or analyzed during the current study are available in the Open Datasus website repository, [https://opendatasus.saude.gov.br/organization/ministerio-da-saude].
